# Notes of Travel in Mexico

**Published:** 1905-01

**Authors:** Herbert U. Williams

**Affiliations:** Buffalo, N. Y., Professor of Pathology, University of Buffalo.


### Notes of Travel in Mexico.

By HERBERT U. WILLIAMS, M. D., Buffalo, N. Y.,
Professor of Pathology, University of Buffalo.

I SPENT six weeks in Mexico last fall, and made some notes on my experiences for the Journal. As I went away for a vacation and a change, I rather avoided medical problems. However, I learned a few things that may interest physicians in our part of America. While I saw nothing of medical work, the physicians told me some things of interest.

There are some five or six thousand English speaking people in the city of Mexico, most of whom came from the United States.

A newspaper in English, the *Mexican Herald,* is published there, which is a member of the Associated Press and is a first-class American paper. Mexico also has an American club and a British club, managed exactly like clubs in this country, and where one would never imagine he was two or three thousand miles from home. There are some very good American physicians in the city. It is considered perfectly proper for them to publish their cards in the *Mexican Herald.* The Americans (meaning the people from the United States) maintain a good modern hospital of about forty beds, and having American trained nurses. I was told that there is a lack of American nurses to do private nursing and that they are paid ten dollars a day, which is nearly five dollars in our money. Proprietary medicines are used, as they are all over the world, if one may judge from the advertisements. It is curious to see familiar signs in the trolley cars, but translated into Spanish, as

"EL PAIN KILLER
DE PERRY DAVIS."

It was my good fortune to meet a number of American physicians, among them Dr. E. H. Norton, for several years surgeon to the Fitch Hospital in Buffalo. His friends will be glad to know that he is well and is prospering.

As the city of Mexico is more than seven thousand feet above sea-level, the climate is temperate. During the two weeks I spent there early in September, the thermometer did not go above 73°. Medical experience seems to be of about the same character as here. Pneumonia, I was told, is not very common but is extremely fatal, presumably on account of the high altitude. Malaria is said to occur in the city of Mexico, but I should like to see the diagnosis proved by blood examination. The Mexican physicians state that the anopheles mosquito, as well as the stegomgia or yellow fever mosquito, is not found in the city.[1]

One disease appears there which is never seen here, and that is typhus fever. It was stated to me that there were three hundred cases in the city last year. Typhus is rare, however, except among the Mexicans. Apparently it does not prevail in the tropical parts of the country, but rather in the winter season and at high altitudes through the center of Mexico, as far north as Zacatecas (altitude about 8,000 feet). This is noteworthy, because typhus in some respect resembles the so-called spotted fever of the Rocky Mountains, which has been described in Wyoming, Montana, Nevada, and Idaho.[2]

---

1. Ruiz. American Public Health Association Reports, Vol. XXIX., 77.

2. See the article of Wilson and Chowning. *Journal of Infectious Diseases*, Chicago, January, 1904.

Of the Mexicans, I saw no more than may any casual traveler. Their picturesque dress and appearance make one think almost that he is looking at a scene out of light opera, rather than real life. Roughly speaking, it seemed to me that nine-tenths of the people one meets through the country are wholly or partly of the American Indian race. Mexicans of the lower class have the reputation of being very dirty in respect to their habits and persons. As far as my experience went, the reputation seems to have been well earned, though I imagine the peasants of a number of European countries are as bad. Certainly, the traveler in Mexico finds fleas, bugs, and their kindred, very troublesome.

About half of my time was spent in the Sierra Madre Mountains in the northwestern part of the country, where the states of Chihuahua and Durango join. As our party traveled with a pack train, we had an opportunity to see something of the Mexicans of the ranches. When our *mozo* (hired man) was once given the dishes to clean, he carefully washed his hands, which were very dirty, with soap and warm water. Then he washed the dishes in the same water. We gave up trying to reform him, and relegated him to packing the mules, in which art he excelled.

On this trip we had a chance to verify some of the stories told of what these Mexicans can endure in the way of exposure. When I started, I heard an old prospector say, "why, I have slept out in the mountains and nearly frozen under six blankets and my *mozo* seemed to be warm enough under a coffee-sack." We camped at an altitude of 9,000 feet, and often found it chilly in a tent with plenty of bedding. But our *mozos* seemed to be satisfied to sleep under a tree, even if it rained, though they were barefooted, poorly clad, and had only a couple of thin damp blankets. The dwellings of the poor people are made of adobe or of logs, with damp and nasty earth for a floor. It seemed to me that many of them suffered from catarrhs. Certainly they were perpetually blowing their noses, coughing and expectorating. Nevertheless, the climate at these high altitudes is superb. The air is so clear that mountains twenty to fifty miles away look as though they were standing out of a relief map. We heard a good deal of rattlesnakes, tarantulas, scorpions, and centipedes until we got on the ground, but then we learned that no one pays much attention to them. Probably they are more troublesome in the low, hot parts of the country. Many of the Mexicans certainly go bare-legged, and wear sandals or nothing at all on the feet.

Although we were in a region said to be infested with rattlesnakes all of our party together killed only six. They were of two different species—the diamond back *(crotalus adamanteus)* which we found large and sluggish, and another totally different species, quite small, with a spotted skin and vicious. One of the diamond-backs was very large and thick, and the body was distended with two or three big bunches, presumably rats or gophers he had eaten. As I wished to photograph him, our American guide resorted to the cowboy's invariable expedient and "roped" him just back of the head with a piece of twine. The snake made very little resistance and acted as though he had taken a narcotic. We made many inquiries concerning the mortality from snake-bites, but could find only a few people who had personally known of anyone even being bitten. From what I had heard of rattle-snakes in New York and Pennsylvania, I had gathered quite a different impression. The pharmacist in a large mining town in the part of Mexico where we were, stated that in the two years of his experience he had never had a call for a remedy for snake-bite. A mine superintendent reported that he had once known a Mexican to die quickly from the bite of a very large rattlesnake,—the only fatal case I heard of. An American trapper told the story of a man who nearly died of a bite on the arm, and who subsequently suffered from redness and swelling of the arm annually at the time of the year when he was first bitten. According to Langmann, there have been cases of this sort that are well authenticated.[1]

One of the most novel experiences we had in the Sierra Madres came in visiting a small settlement of Tarahumare Indians. This tribe by reason of living in wild and remote parts of the mountains, often in deep canons (also called *barrancas* in Mexico), has been less affected by contact with white men than most of the Indians in the United States. The Tarahumares have been very carefully described by Dr. Carl Lumholz (Unknown Mexico, *Scribner's*, 1902,) who studied there for several years. Some of those that we saw wore clothes, but others had no garment but a breech-cloth. They had no weapons but bows and arrows, with which they shot parrots and other birds, squirrels, and rarely deer. We found an arrow sticking in the soil by a pond, where some Indian had probably been hunting ducks. They cultivate corn, and secure some very good crops. One day we encountered unexpectedly a young fellow from this tribe, and I shall not forget the impression made by seeing a wild man in his native wild environment. He was stark naked but for his breech-cloth. His hair was a little wavy, and came well down on his neck. His teeth were glistening white, and perfect in form. His bearing was quiet and dignified. When he parted from us he went off at an easy, graceful lope, making about five miles an hour, regardless of rocks and logs, carrying his bow and arrows in his hands.

1. Langmann. Reference Handbook of the Medical Sciences, 1903. VI., 712.

He was built for speed, with slim arms and legs, every muscle perfect, and with a splendid back and chest and a mass of pectoral muscle. He reminded me of nothing so much as Kipling's Mowgli. We heard the most astonishing tales of the endurance of these people in running, but they are fully verified by Lumholz. He even makes the almost incredible statement that a Tarahumare will easily run one hundred and seventy miles without stopping. One of them carried a load of one hundred pounds on his back, a distance of one hundred and ten miles in seventy hours. All over Mexico, indeed, one sees the natives working as beasts of burden, along with the burros and mules, and bearing tremendous loads.

A gentleman who had had a great deal of surgical experience among the Indians of Mexico, told me that their capability for enduring pain was extraordinary. He had seen them bear the most shocking injuries with stolidity, and submit to terrific operations without an anesthetic. In fact, they preferred not to take an anesthetic. Lumholz relates that he once pulled six hairs at a time from the head of a sleeping child without disturbing it, and when twenty-three hairs were pulled out in one stroke, the child only scratched its head a little and slept on.

I appreciate that these notes are disconnected and mostly based on what was told me in conversation. But, as I remarked in beginning, I was not on the lookout for facts related to medical work. As to the other aspects of Mexico there is only space here to say that it is a country of immense distances, with many things that are novel, interesting, and beautiful.

221 North Street.

---

### THE HISTORY OF TUBERCULOSIS.

John B. Huber (*Medical Record,* October 22, 1904,) gives an interesting resume of the history of tuberculosis and of the views of older physicians concerning its nature, together with some of the sanitary regulations that arose from their conception of the disease. In spite of its less dramatic form, the ravages of this plague are much more dreadful than those of cholera, smallpox, or the black death, and yet, owing to its slow and insidious course, it causes none of the consternation attending the outbreak of those scourges. The disease has not been without its influence in art and literature, and an interesting list is given of personages eminent in various walks of life who have succumbed to the Great White Plague.

